# The **S**wedish initiative for the st**u**dy of **Prim**ary sclerosing cholangitis (SUPRIM)

**DOI:** 10.1016/j.eclinm.2024.102526

**Published:** 2024-03-11

**Authors:** Martin Cornillet, Christina Villard, Fredrik Rorsman, Antonio Molinaro, Emma Nilsson, Stergios Kechagias, Erik von Seth, Annika Bergquist

**Affiliations:** a Department of Medicine Huddinge, Karolinska Institutet, Stockholm, Sweden; b Department of Transplantation Surgery, Karolinska University Hospital, Stockholm, Sweden; c Department of Gastroenterology and Hepatology, Akademiska University Hospital, Uppsala, Sweden; d Department of Hepatology, Sahlgrenska University Hospital, Göteborg, Sweden; e Gastroenterology Clinic, Skåne University Hospital, Sweden; f Department of Health, Medicine and Caring Sciences, Linköping University, Linköping, Sweden; g Department of Upper Abdominal Diseases, Karolinska University Hospital, Stockholm, Sweden

**Keywords:** Primary sclerosing cholangitis, Inflammatory bowel disease, Liver transplantation, Phenotype, Outcome

## Abstract

**Background:**

Despite more than 50 years of research and parallel improvements in hepatology and oncology, there is still today neither a treatment to prevent disease progression in primary sclerosing cholangitis (PSC), nor reliable early diagnostic tools for the associated hepatobiliary cancers. Importantly, the limited understanding of the underlying biological mechanisms in PSC and its natural history not only affects the identification of new drug targets but implies a lack of surrogate markers that hampers the design of clinical trials and the evaluation of drug efficacy. The lack of easy access to large representative well-characterised prospective resources is an important contributing factor to the current situation.

**Methods:**

We here present the SUPRIM cohort, a national multicentre prospective longitudinal study of unselected PSC patients capturing the representative diversity of PSC phenotypes. We describe the 10-year effort of inclusion and follow-up, an intermediate analysis report including original results, and the associated research resource. All included patients gave written informed consent (recruitment: November 2011–April 2016).

**Findings:**

Out of 512 included patients, 452 patients completed the five-year follow-up without endpoint outcomes. Liver transplantation was performed in 54 patients (10%) and hepatobiliary malignancy was diagnosed in 15 patients (3%). We draw a comprehensive landscape of the multidimensional clinical and biological heterogeneity of PSC illustrating the diversity of PSC phenotypes. Performances of available predictive scores are compared and perspectives on the continuation of the SUPRIM cohort are provided.

**Interpretation:**

We envision the SUPRIM cohort as an open-access collaborative resource to accelerate the generation of new knowledge and independent validations of promising ones with the aim to uncover reliable diagnostics, prognostic tools, surrogate markers, and new treatment targets by 2040.

**Funding:**

This work was supported by the 10.13039/501100002794Swedish Cancer Society, Stockholm County Council, and the Cancer Research Funds of Radiumhemmet.

**Research in context**

**Evidence before this study**

Primary sclerosing cholangitis (PSC) is a rare cholestatic liver disease, recognized as one of the biggest unmet needs in hepatology. Previous research established that PSC patients have highly heterogeneous clinical phenotypes and progressions. Together with the lack of large representative prospective biobank clinically annotated, this has hampered the pathogenesis understanding of the disease, the discovery of diagnostic and prognostic tools and treatments targets.

**Added value of this study**

We provide descriptions and results from a large national multicenter prospected study (SUPRIM), associated clinical data and biological samples. The SUPRM cohort represents the heterogeneity of clinical phenotypes and progressions in PSC and constitutes a unique resource for research.

**Implications of all the available evidence**

We envision the SUPRIM cohort as an open-access collaborative resource to accelerate the generation of new knowledge and independent validations of promising ones with the aim to uncover reliable diagnostics, prognostic tools, surrogate markers, and new treatment targets by 2040.

## Introduction

First described one hundred years ago,^1^ primary sclerosing cholangitis (PSC) is a rare liver disease. The clinical presentation and progression in PSC are highly heterogeneous.^2^, ^3^, ^4^, ^5^, ^6^ PSC can lead to various degree of fibrosis and multifocal biliary strictures, often over the course of several decades. It can affect both sexes but is typically diagnosed in males in their 30s, although the age at diagnosis spans from childhood^7^^,^^8^ to late adulthood.^9^ A large subset of patients has inflammatory bowel disease (IBD), more frequently ulcerative colitis (UC) than Crohn's disease (CD) harbouring specific features.^10^ Upon PSC progression, most of the patients might ultimately need a liver transplantation (LT).^11^ Although the median time to LT is around 20 years, some patients progress more rapidly.^12^ A wide spectrum of unpredictable clinical complications can arise including cholangiocarcinoma (CCA), which has a dismal prognosis.^13^^,^^14^ Finally, the heterogeneous clinical presentation and/or progression of PSC can additionally be ascribed to the involvement of small or large bile ducts, the presence of features of autoimmune hepatitis or the liver-related biochemical profile.^14^, ^15^, ^16^, ^17^, ^18^

Despite more than 50 years of research and tremendous parallel improvements in hepatology and oncology, there is still today neither a treatment to prevent disease progression in primary sclerosing cholangitis (PSC) nor a reliable early diagnostic for the associated hepatobiliary cancers. Importantly, the limited understanding of the underlying biological mechanisms in PSC and its natural history not only affects the identification of new drug targets but implies a lack of surrogate markers that hampers the design of clinical trials and the evaluation of drug efficacy.^19^ In a similar time-lapse, progress has been made at a much faster pace in related fields, and the recent Nobel Prizes in Medicine for cancer immunotherapy (2018) and hepatitis C (2020) are witnesses of such achievements. Given that PSC is a rare heterogenous disease, and in the light of such contrasted success stories, the dogmatic liver-centric research might gain to be broaden. Lessons from ongoing successful developments and implementations in other fields must be seen as a new change when the hepatic disease burden is changing.^20^ Today, non-invasive sampling is not anymore only providing biomarkers but also opportunities to capture and understand disease pathogenesis in a more holistic way. A paradigm shift is operating as non-invasive sampling is replacing biopsies for diagnostics, prognosis, treatment stratification and monitoring of responses to therapies.^21^, ^22^, ^23^ Non-invasively collected biological material such as plasma, serum or blood is becoming standard of care for practical and scientific reasons. They present benefits in terms of feasibility, are less subject to bias induced by selective regional biopsies and thus better capturing the biological heterogeneity and are more compatible with longitudinal monitoring. In summary, the analysis of the so-called “circulome” by “liquid biopsies” represents a key avenue to couple research discoveries with implementable tools. Among the most established methods are the detection of circulating entities such as extracellular vesicles or cells, and analysis of associated or free circulating biological material. The progress of coverage, modalities, and sensitivity of downstream analytical pipelines such as proteomics, metabolomics, and genomics offer a growing range of complementary layers of information. One can mention the progress in the detection of circulating genetic material (such as DNA, miRNA, or lncRNA) followed by related genetic (such as mutations, deletions, translocations, or amplifications) or epigenetic analysis (such as methylation, hydromethylation, or histone modification).^24^^,^^25^ Another key aspect is proteomics, that is now offering a new world to be explored (high coverage, post translational modifications, isoforms analysis, antibodies).^26^^,^^27^ Together with the improvement of comprehensive associated reference maps of the human body, cell types and pathways,^28^, ^29^, ^30^ accessible analysis tools including integrative methods and artificial intelligence (AI),^31^, ^32^, ^33^ we can anticipate that such approaches can generate a deeper understanding of PSC.

As a possible platform for such implementation, we describe the SUPRIM cohort, a decade of clinical data and biological samples collection that we would like to make available for cutting edge research. Several areas of research are of specific interest in PSC, thus possibly relevant to better understand its pathogenesis, but also to transfer back the knowledge gained. PSC is a model of opportunity to study the carcinogenesis processes of CCA in the Western world and to develop and validate early diagnostic tools for cancer. It offers various phenotypes such as autoimmune hepatitis (AIH)–and IgG4-high variants that can be studied in relation to similar disorders to dissect common features and specificities. It touches upon intricated links with autoimmune diseases and IBD offering potential synergy for knowledge transfer and drug repurposing.^34^ Finally, the study of sex specificities has a growing appreciated importance and might reveal important aspect of the disease pathogenesis.^35^, ^36^, ^37^

In this context, we envision the SUPRIM cohort as an open-access collaborative resource to address these knowledges gaps and improve the management of patients. All fields of research are encouraged to be involved and we can collectively hope that this will lead to breakthrough in PSC research, with findings benefiting to other related diseases.

## Methods

### Participants and settings

This study was performed with two aims; to evaluate the efficacy of yearly MRI surveillance in PSC for early detection of hepatobiliary malignancy (41), and to describe the natural history and collect a large biobank prospectively in PSC, presented here. Eleven Swedish centres recruited PSC adults between 1st November 2011 and 1st April 2016 and followed them for 5 years, Supplementary Table S1. Inclusion criterion was a diagnosis of PSC, based on accepted diagnostic criteria with cholestatic liver biochemistry with typical cholangiographic features on MRCP and/or a liver biopsy.^6^ Exclusion criteria were previous LT, expected listing for LT within one year after inclusion, a diagnosis of hepatobiliary malignancy at time of inclusion and lack of a baseline MRI/MRCP. At the time of inclusion, data on age, sex, comorbidities, previous medical history, IBD, medications, liver function tests, tumour markers and imaging (MRI/MRCP) were collected.

Data was registered by the treating physician in an electronic clinical report form. At yearly follow-ups in patients without endpoints, clinical data including symptoms, medication, imaging, endoscopic interventions, and results of MRI/MRCPs and colonoscopies were registered. Yearly blood sample collections included liver functions tests (bilirubin AST, ALT, GGT, AP, albumin, INR) and cancer associated antigen 19-9 (CA19-9). Patient were followed until LT, hepatobiliary cancer or CCA. Indications for LT were end-stage liver disease or perihilar high-grade dysplasia.^5^ The complete study protocol is found in supplementary material. All patients with a positive AMA were reviewed in detail. PBC was ruled out by a liver biopsy in nine out of 12 patients. In the other three patients, the PSC and IBD were diagnosed at an early age and there was no data in the hospital charts from the time describing whether a biopsy had been performed.

### Ethics statement

The regional Ethics Committee in Stockholm approved the study (Dnr 2011/824-31/2), clinicaltrials.gov NCT03041662. All included patients gave written informed consent.

### Definitions

PSC and IBD was defined according to the treating physician using accepted diagnostic criteria.^5^^,^^6^^,^^38^ Presence of AIH and small duct PSC was defined based on the treating physician's clinical evaluation in line with clinical guidelines,^5^^,^^6^ cirrhosis was diagnosed on a biopsy or when radiology signs indicative of cirrhosis were present. Data on jaundice intervention i.e. ERCP was collected from the Swedish Registry of Gallstone Surgery and Endoscopic Retrograde Cholangiopancreatography (GallRiks).^39^ The cut off for IgG4 high was >2 g/L. Progression of biliary stricturing was based on the MRI reports and categorized as either stable or severe/progressive bile duct changes.

The study endpoints were a diagnosis of hepatobiliary malignancy, liver transplantation due to end-stage liver disease or severe complications of cholangitis and death in patients with PSC during 5-year follow-up. Indications for LT were end-stage liver disease or perihilar high-grade dysplasia.^5^

### Statistical analysis

Descriptive information at baseline for the cohort is presented as median (IQR) for continuous variables and n (%) for categorical variables. Patients were followed for five years, or to the date of death or LT. Cox proportional hazards regression analysis was used to determine factors associated with time to LT. Baseline variables were initially analysed in a univariate regression analysis. Any variable with a p value < 0.1 was included in a multivariable regression model. The dates for blood sampling were treated as time-varying in the Cox regression analysis, allowing several observational periods per patient. The result from each visit, including the predictive scores, was a predictive factor for outcome during that year, e.g. until the next visit or outcome, whichever came first. Clustered robust standard errors were used to adjust for the intra-person correlation caused by having several observed time intervals per participant in the Cox regression analysis. Fluctuations of liver function tests and predictive scores were assessed using a mixed model with person-specific random intercept. Each timepoint was treated as a separate row, allowing all information to be included in the model. Validation of the score models: revised Mayo risk score (MRS), Amsterdam–Oxford score and the Primary Sclerosing Cholangitis Risk Estimate Tool (PREsTO) was performed by using the Harrell's concordance index (C-statistics) and Sommers D were used for discrimination analyses. Statistical significance was assumed for p values < 0.05. Statistical analysis was performed using STATA 17.

### Role of the funding source

Funding for the present study was used for salaries of physicians/clinical researchers and research staff for biobanking and entering clinical data to the case report forms. The funders were not involved in study design, data analysis or manuscript writing. All Authors (Martin Cornillet, Christina Villard, Fredrik Rorsman, Antonio Molinaro, Emma Nilsson, Stergios Kechagias, Erik von Seth, Annika Bergquist) have access to the database and the decision for publication was taken in consensus.

## Results

### Clinical characteristics capturing the representative diversity of PSC phenotypes and disease progression

We here present the SUPRIM cohort, a national prospective study, a 10-year effort of inclusion, follow-up, data, and sample collection. The intermediate analysis report includes original results, and the associated research resource. Many of these patients (and additional ones) are still undergoing structured follow up with standardised data collection and biobanking by inclusion in the PiSCATIN study, a phase III randomized double-blind controlled trial of 40 mg simvastatin versus placebo,^40^ as continuation of the SUPRIM cohort but will not be described in detail in the current report.

The design of the SUPRIM cohort (unselected, prospective, and longitudinal) aimed to capture the representative diversity of the PSC phenotypes and evaluate the efficacy of MRI surveillance for early detection of CCA.^41^ Five-hundred and twelve consecutive PSC patients from 11 Swedish hospitals were included. The cohort is representative to other cohorts with PSC with a median age at inclusion of 38 years [IQR 19] and a median duration of PSC of seven years [IQR 11]. The majority were males (345, 68%) had IBD (397, 79%) and 326, 64% were treated with ursodeoxycholic acid (UDCA). PSC and IBD characteristics, medications and liver function tests, predictive score and endpoint outcomes are presented in (Table 1). Out of 512 included patients, 452 patients completed the five-years follow-up without endpoint outcomes. LT was performed in 54 patients (10%), hepatobiliary (HB) malignancy was diagnosed in 15 patients (3%) and colorectal cancer/high grade dysplasia in 11 of 322 PSC-IBD patients (3%). The primary indication for LT was end-stage liver disease in 45 patients (83%) and biliary dysplasia in 9 patients (17%). Thirty-two patients (6%) had small duct PSC, 57 patients (11%) had PSC with features of AIH and elevated IgG was observed in seven patients (1%). The majority had normal or mildly elevated and stable liver function tests as shown in Table 1 and Supplementary Figure S1. Features associated with LT due to hepatic decompensation and/or severe complications of cholangitis in patients with PSC during five-year follow-up are presented in (Table 2). As expected, LT was associated with liver function tests, jaundice, progressive bile duct changes on MRI and bacterial cholangitis.

**Table 1:** Patients’ characteristics during five-year follow-up.

	Inclusion N = 512	Year 1 N = 497	Year 2 N = 485	Year 3 N = 477	Year 4 N = 459	Year 5 N = 452
Clinical appointment	512 (100)	491 (99)	473 (98)	444 (93)	408 (89)	342 (76)
Blood sample collection	512 (100)	488 (98)	462 (95)	434 (91)	385 (84)	312 (69)
Imaging (MRI/MRCP)	512 (100)	443 (89)	432 (89)	402 (84)	371 (81)	303 (67)
Events						
ERCP	25 (5)	67 (13)	59 (12)	46 (10)	27 (6)	29 (6)
Colonoscopy	361 (71)	294 (59)	284 (56)	249 (52)	227 (49)	179 (40)
Hepatobiliary malignancy	4 (1)	4 (1)	1 (0.5)	4 (1)	2 (0.5)	0
Colorectal malignancy (including hgd)	3 (1)	4 (1)	1 (0.5)	1 (0.5)	1 (0.5)	1 (0.5)
Liver transplantation	13 (3)	8 (2)	6 (1)	8 (2)	2 (0.5)	17 (4)
Deceased	2 (0.5)	7 (1)	2 (0.5)	6 (1)	3 (1)	5 (1)
General characteristics						
Male gender	345 (68)					
Age	38 [19][14][1]	39 [19][14][1]	40 [19][14][1]	41 [19][14][1]	43 [19][14][1]	43 [19][14][1]
BMI	24 [4][4][0]	24 [5][4][0]	24 [5][4][0]	25 [6][5][0]	25 [5][5][0]	25 [6][5][0]
PSC characteristics						
Small duct PSC	32 (6)					
Features of AIH	57 (11)					
Duration of PSC	7 [11]	8 [11]	9 [11]	10 [11]	11 [11]	12 [11]
Cirrhosis	59 (12)					
Ascites	4 (1)	7 (1)	11 (2)	13 (3)	7 (2)	5 (2)
Treatment of cholangitis	33 (7)	26 (5)	30 (7)	30 (7)	24 (6)	16 (5)
Jaundice intervention	25 (5)	18 (4)	18 (4)	14 (3)	13 (3)	7 (2)
Treatment of bleeding varices	6 (1)	7 (1)	9 (2)	5 (1)	6 (1)	2 (1)
Previous episode of encephalopathy	3 (1)	7 (1)	9 (2)	7 (2)	6 (2)	7 (2)
Progression of biliary stricturing on imaging	43 (8)	40 (9)	42 (10)	35 (9)	26 (7)	26 (9)
IBD						
Ulcerative colitis	322 (64)					
Crohns disease	75 (15)					
Duration of IBD	15 [16][12][1]	16 [16][12][1]	17 [16][12][1]	18 [16][12][1]	19 [16][12][1]	20 [17][12][1]
Colectomy	79 (20)					
Medication						
Ursodeoxycholic acid (UDCA)	326 (64)					
5-aminosalicylic acid (5-ASA)	344 (70)					
Liver function tests						
Bilirubin (μmol/l)	11 [8][18][0.8]	12 [11][17][1]	11 [9][16][1]	11 [9.3][23][1]	11 [8][37][2]	11 [9][16][1]
Alkaline phosphatase, ALP (U/L)	138 [204][173][8]	138 [204][195][9]	138 [186][199][9]	132 [192][171][8]	138 [167][404][21]	132 [174][155][9]
Aspartate aminotransferase, AST (U/L)	41 [54][181][8]	42 [52][198][9]	40 [46][119][6]	39 [48][143][7]	41 [44][319][16]	39 [39][42][2]
Alanine aminotransferase, ALT (U/L)	50 [86][95][4]	52 [85][86][4]	50 [72][113][5]	45 [73][211][10]	48 [70][534][27]	43 [58][59][4]
Gamma-glutamyl transferase, GGT (U/L)	135 [356][399][18]	144 [364][469][22]	150 [334][1198][57]	120 [310][405][20]	132 [338][382][20]	120 [355][438][25]
Albumin (g/L)	39 [6][5][0]	40 [6][4][0]	39 [5][5][0]	38 [5][5][0]	39 [5][4][0]	39 [5][4][0]
International normalized ratio (INR)	1.0 [0.1][0.3][0]	1 [0.2][0.2][0]	1 [0.2][0.2][0]	1 [0.2][0.2][0]	1 [0.2][0.2][0]	1 [0.2][0.2][0]
Platelets (10^9^/L)	244 [105][94][4]	240 [112][97][4]	238 [103][89][4]	238 [109][89][4]	235 [104][91][5]	242 [104][90][5]
Creatinine (mmol/L)	72 [19][16][1]	73 [20][16][1]	74 [20][21][1]	74 [20][18][1]	76 [19][22][1]	75 [20][18][1]
Immunoglobins (g/L)						
Immunoglobin total	12.8 [4.4][4.7][0.2]					
Immunoglobulin G4 (IgG4)	0.3 [0.4][0.5][0.3]					
IGg4 high (>2)	7 (2)					
Immunoglobulin A	2.3 [1.5][14.2][0.7]					
Autoantibodies						
Positive antinuclear antibodies (ANA)	108 (24)					
Anti-mitochondrial antibodies (AMA)	1264 (3)					
Smooth muscle antibodies (SMA)	61 (14)					
Autoimmune diseases						
Thyroid	27 (5)					
Skin	26 (5)					
Other	46 (9)					
Prognostic scores						
MELD score	7 [2][2][0]	7 [2][2][0]	7 [2][2][0]	7 [2][3][0]	7 [2][3][0]	6 [2][2][0]
Revised mayo score^a^ (score and equivalent percentage of 4- year survival)	−0.2 [1.2][0.9][0] (low risk, >94%)	−0.2 [1.2][0.9][0] (low risk, >94%)	−0.1 [1.2][1.0][0] (low risk, >94%)	−0.1 [−1.1][1.0][0] (low risk, >94%)	−0.1 [1.2][0.9][0] (low risk, >94%)	−0.1 [0.9][0.9][0.1] (low risk, >94%)
Amsterdam–oxford score (5-year transplant free estimated survival, %)	93 [6][8][0]	92 [6][10][1]	92 [5][12][1]	92 [6][8][0]	92 [5][9][1]	92 [5][9][1]
PREsTO score (5-year probability of decompensation, %)	3.9 [4.0][10.3][0.5]	4.3 [4.5][11.8][0.6]	4.4 [5.4][13.1][0.6]	4.5 [4.9][13.8][0.7]	4.4 [5.0][10.8][0.6]	4.7 [5.4][11.5][0.7]

Data presented in median [interquartile range], [SD] [SEM] for continuous variables and frequency (percent) for categorical variables.

a This score predicts 4 years survival in individual patients.

**Table 2:** Features associated with liver transplantation due to end-stage liver disease or severe complications of cholangitis in patients with PSC during 5-year follow-up, assessed by cox proportional hazards regression.

	Univariate analysis HR (95% confidence interval)	p-value	Multivariate analysis HR (95% confidence interval)	p-value
Male sex	1.62 (0.82–3.26)	0.172		
Age	1.00 (0.98–1.02)	0.849		
Body mass index (BMI) (at inclusion)	0.92 (0.80–1.05)	0.193		
Severity of liver disease				
Small duct PSC	1.01 (0.31–3.22)	0.983		
PSC with autoimmune features	1.60 (0.77–3.26)	0.210		
Duration of PSC (at inclusion)	1.04 (1.01–1.07)	0.004		
Ascites	1.03 (3.73–24.55)	**<0.001**	0.20 (0.002–19.50)	0.492
Treatment of cholangitis	11.31 (5.63–22.71)	**<0.001**	7.85 (1.43–43.07)	**0.018**
Jaundice intervention	8.99 (3.91–20.67)	**<0.001**	6.40 (1.66–24.66)	**0.007**
MRI/MRCP with severe/progressive bile duct changes	4.85 (2.32–10.16)	**<0.001**	7.42 (2.02–27.22)	**0.003**
Cirrhosis (at inclusion)	4.08 (2.25–7.40)	**<0.001**	0.20 (0.02–1.81)	0.152
Inflammatory bowel disease (IBD)				
Diagnosis of IBD	0.73 (0.37–1.44)	0.369		
Ulcerative colitis	1.17 (0.63–2.16)	0.619		
Crohns disease	0.53 (0.19–1.49)	0.231		
Duration of IBD (at inclusion)	0.98 (0.96–1.01)	0.200		
Colectomy (at inclusion)	0.74 (0.29–1.98)	0.525		
Medications				
Ursodeoxycholic acid (at inclusion)	2.22 (1.08–4.60)	**0.031**	1.47 (0.23–9.36)	0.681
5-aminosalicylic acid (5-ASA) (at inclusion)	0.84 (0.35–1.17)	0.146		
Tumour markers				
Carbohydrate antigen 19-9 (CA19-9)	1.00 (1.00–1.01)	**<0.001**	1.00 (1.00–1.01)	**0.004**
Carcinoembryonic antigen (CEA)	1.05 (1.01–1.10)	**0.014**	1.01 (0.84–1.22)	0.878
Liver function tests				
Bilirubin (Μmol/L)	1.03 (1.02–1.03)	**<0.001**	1.02 (1.00–1.05)	0.059
Alkaline phosphatase, AP (U/L)	1.19 (1.13–1.25)	**<0.001**	1.12 (0.81–1.53)	0.498
Aspartate aminotransferase, AST (U/L)	1.04 (1.02–1.07)	**<0.001**	1.03 (0.96–1.11)	0.373
Alanine aminotransferase, ALT (U/L)	1.06 (1.03–1.09)	**<0.001**	0.66 (0.33–1.32)	0.239
Albumin (g/L)	0.88 (0.84–0.91)	**<0.001**	0.92 (0.71–1.19)	0.517
International normalized ratio, INR	3.79 (3.05–4.73)	**<0.001**	0.04 (0.00–0.96)	**0.047**
Platelets (109/L)	0.98 (0.98–0.99)	**<0.001**	0.99 (0.98–1.00)	0.200
Immunoglobins (g/L)				
Immunoglobulin total (at inclusion)	1.09 (1.05–1.13)	**<0.001**	1.09 (1.01–1.18)	**0.023**
Immunoglobulin G4 (IgG4) (at inclusion)	1.06 (0.62–1.82)	0.817		
IgG4 high (>2)	1.96 (0.24–15.76)	0.526		
Immunoglobulin A (IgA) (at inclusion)	1.00 (0.98–1.01)	0.561		
Autoantibodies				
Positive antinuclear antibodies (ANA)	1.00 (0.50–1.97)	0.978		
Anti-mitochondrial antibodies (AMA)	1.00 (0.97–1.02)	0.375		
Smooth muscle antibodies (SMA)	1.48 (0.70–3.09)	0.303		
Prognostic scores				
MELD	1.25 (1.21–1.29)	**<0.001**	1.60 (1.25- 2–05)	**<0.001**
Revised Mayo score	3.98 (3.10–5.12)	**<0.001**	0.100 (0.38–2.64)	0.997
Amsterdam–Oxford score	4.73 (3.03–7.36)	**<0.001**	0.98 (0.93–1.04)	0.594
PREsTO	1.05 (1.04–1.06)	**<0.001**	1.90 (0.36–10.10)	0.452

Bold denotes p > 0.05.

Overall, the SUPRIM cohort captured the representative diversity of PSC phenotypes and progressions. It consequently offers an important resource not only to understand the disease spectrum, but also to develop and validate tests in a realistic setting.

### The multidimensional clinical landscape of PSC underscores heterogeneity of the disease

PSC is often described as a disease affecting young males with IBD. However, this reductionistic picture doesn't represent the heterogeneity of the disease. A risk might exist, to focus an excessive attention studding a very narrow non-representative phenotype with the aim to extrapolate the biological findings to the rest of the spectrum. First, subgroups of PSC have been suggested to have distinct pathophysiology, such as “overlap AIH” and “small duct PSC”.^5^ In addition, time is a parameter to consider in three dimensions since the age at diagnosis, the age at the time of study, and the time spent from diagnosis are important aspects of the disease course. Furthermore, all combinations of sex and IBD are part of the PSC continuum. Finally, the presence of cirrhosis is a critical time dependent component of the disease.

**Fig. 1:**
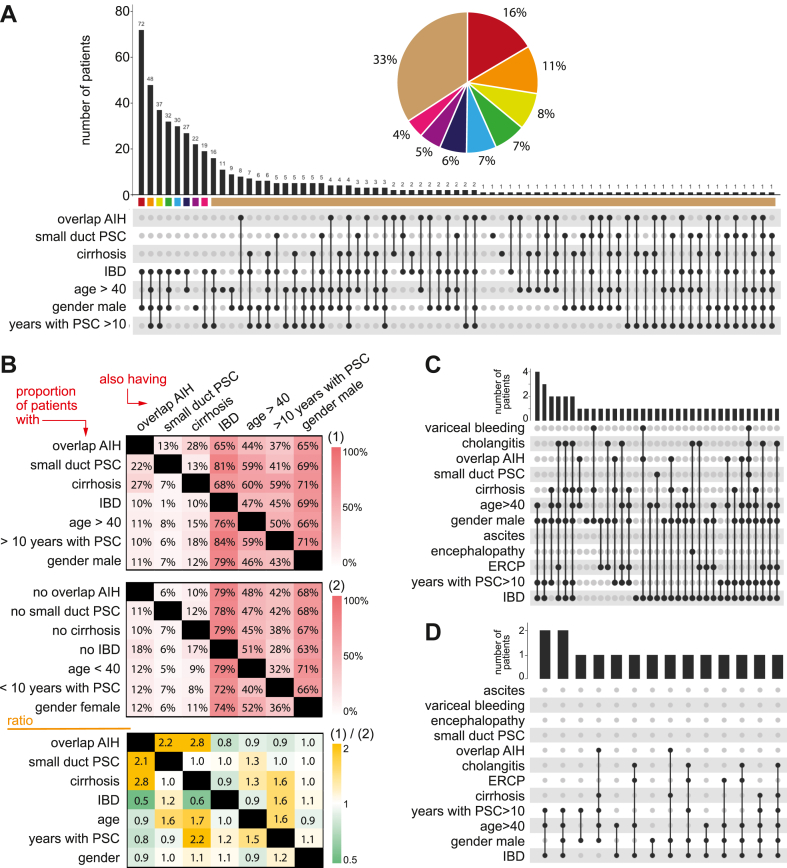
Multidimensional clinical landscape of PSC at baseline. This figure illustrates the complexity and heterogeneity of the clinical presentation of PSC. (A) Baseline characteristics of the SUPRIM cohort. Combinations of features are depicted by black filled circles and the corresponding number of patients harboring every combination of feature is indicated above with histograms. For example, the proportion of patients in this cohort with cirrhosis that also have overlap with AIH is 27% and the proportion of men having IBD is 79%. (B) Proportions of all paired associations of features at baseline. (C) Baseline clinical characteristics at inclusion of the study of the subgroup of patients transplanted during the 5 years follow up. The symptom pattern varies a lot and variceal bleeding, cholangitis and endoscopic intervention with ERCP were among the most common symptomatic features. (D) Baseline clinical characteristics at inclusion of the study of the subgroup of patients that developed hepatobiliary malignancy during the 5 years follow-up. The majority was older than 40 years.

The clinical landscape of PSC continuum is composed of a large variety of distinct profiles (Fig. 1A). Most of the PSC patients are, as expected, males with IBD, however integrating extra layers of information highlighted that about a third of the continuum is represented by patients with relatively unique/specific phenotypes. Men, younger than 40 years with IBD but no “overlap AIH”, “small duct PSC” or cirrhosis (n = 72), illustrated by the left bar in Fig. 1A, represented a minor part of the PSC continuum. Associations of features is highlighted in Fig. 1B, including the time components in the disease course, as 50% of patients more than 40 years old had had the diagnosis more then 10 years before inclusion. Cirrhosis was significantly associated with PSC with autoimmune features, 2.8-fold increase (Fig. 1B). Finally, the baseline phenotyping of patients being transplanted (Fig. 1C) or diagnosed with HB cancers (Fig. 1D) revealed almost a unique profile per patient, illustrating the difficult challenge to understand the common biological features underlining these conditions.

Overall, associations of multiple layers of important clinical features revealing common and unique traits between patients is captured. The SUPRIM cohort offers an important resource to search for both largely applicable prognostic and diagnostic tests, and personalised medicine strategies.

### Multidimensional biological landscape of PSC

Several biochemical parameters are used worldwide in clinical routine to monitor the disease progression but none of them is specific for PSC. They capture some incomplete but important biological aspects of the disease such as liver damage or inflammation. Understanding the biological relationships both at the global scale (“cohort-scale”), and in a single individual (“patient-scale”) is a challenge and interpretation of results at these two different scales are different with important consequences.

**Fig. 2:**
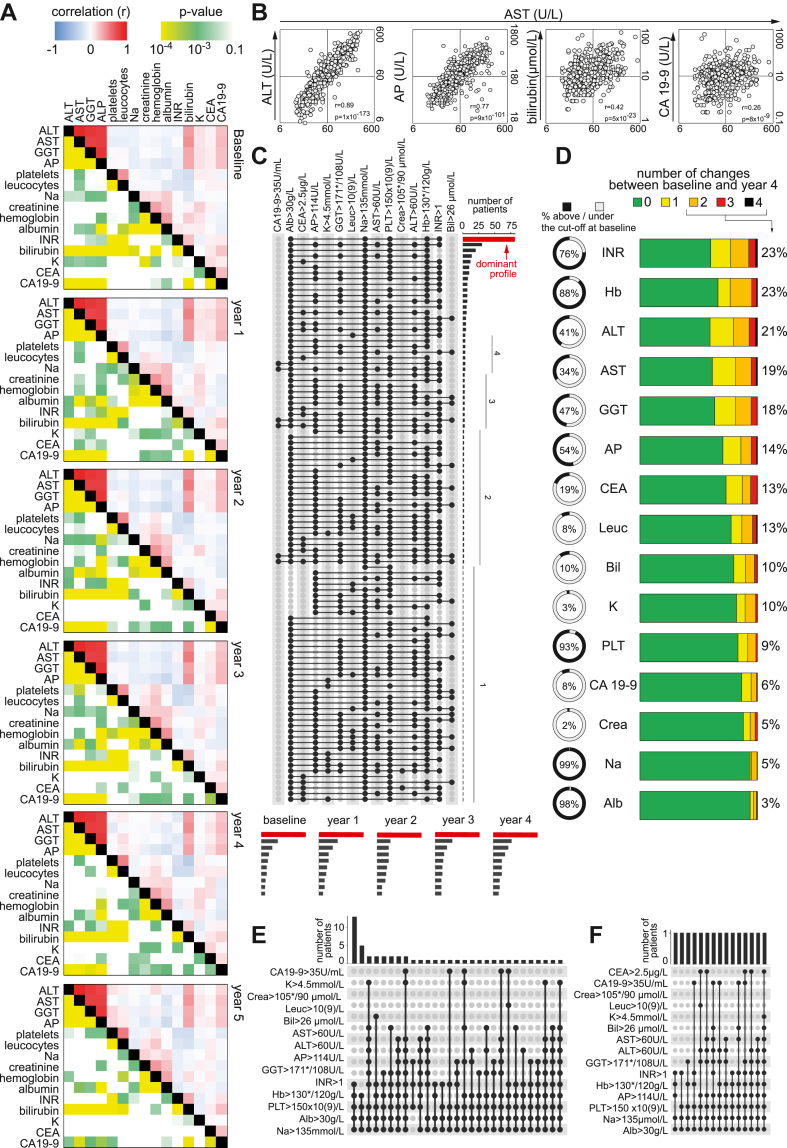
Multidimensional biological landscape of PSC. This figure depicts the complexity and heterogeneity of common clinically used parameters used for following PSC. The whole cohort as well as subgroups are shown. (A) Correlations of biological parameters overtime of the whole SUPRIM cohort. Similar pattern of clinical parameters is seen over time (baseline-year 5). (B) Example of correlations associated r coefficients and p-values. The correlation between AST and ALT, AP is high, as expected, and lower for CA19-9. (C) Baseline characteristics of the SUPRIM cohort. Combinations of features are depicted by black filled circles and the corresponding numbers are indicated above with histograms. The dominant profile of mild disease remains during the 5 years follow up (D) Variability of biological parameters (liver function tests) overtime. (E) Baseline characteristics of the liver function tests for the subgroup of patients transplanted during the 5 years follow up (F) Baseline liver function tests for the subgroup of the patients that developed hepatobiliary malignancy during the 5 years follow-up. ∗ Cut off for male/female. INR: international normalized ratio; Hb: hemoglobin; ALT: alanine aminotransferase; AST: aspartate aminotransferase; GGT: Gamma-glutamyl transferase; AP: alkaline phosphatase; CEA: carcinoembryonic antigen; Leuc: leucocyte; Bil: bilirubin; K: potassium; PLT: platelet: CA19-9: Carbohydrate 19.9 Antigen; Crea: creatinine; Na: sodium; Alb: albumin.

The longitudinal relationships between all biochemical parameters acquired at the cohort-scale showed an extremely stable pattern during a 5-year period (Fig. 2A), underscoring the slow disease progression in most of the patients. As expected, very high and significant correlations between the different liver function tests (ALT, AST, GGT and ALP) were repeatedly observed. These parameters thus appeared almost redundant at the cohort-scale, questioning the need of all four of them, and the need of repeated measurements. However, at the patient-scale one can appreciate both the interindividual variability, outliers, unique patterns, underlining subclinical events at a specific time, in a specific patient (Fig. 2B). The dominant biochemical PSC profile remained identical over the five years period at the cohort-scale (Fig. 2C). However, various degree of fluctuations occurs at the individual scale as summarised in Fig. 2D. Ten %, 14% and 21% of the patients fluctuated (from up to down the thresholds or vice versa) two, three or even four times in their bilirubin, ALP and ALT levels respectively over the period of only 5 measurements. Thus, these two scales provided complementary information to be used for different purposes. Finally, the baseline profiling of patients transplanted or diagnosed with HB cancers (Fig. 2E and F) revealed almost unique profile per patient illustrating once again the challenge to understand the common biological feature underlining these conditions.

Overall, the SUPRIM cohort captured the longitudinal biological variations mirroring the clinical phenotypes and progression in PSC. It offers an important resource to understand the biological–clinical relations to uncover new druggable targets and surrogate markers to evaluate drug efficacy.

### Comparisons of current predictive models in PSC and future needs

The variability in disease progression stresses the need of markers to predict clinical outcomes.^42^ A range of prognostic models have been developed to predict events such as transplant-free survival and hepatic decompensation.^13^^,^^42^, ^43^, ^44^, ^45^, ^46^, ^47^, ^48^, ^49^, ^50^, ^51^ The most broadly used model to assess transplant-free survival by non-invasive predictors is the revised MRS.^44^ More recently additional scores have been developed, among them the Amsterdam–Oxford score and the PREsTO.^45^, ^46^, ^47^ All these models include similar variables but exclude phenotypes differently.^44^, ^45^, ^46^, ^47^ The Amsterdam–Oxford score and PREsTO, for instance, do not include PSC with autoimmune features but sub-group depending on small or large duct PSC, whereas PREsTO neither includes small duct PSC nor PSC with autoimmune features.^45^^,^^47^ None of these score models take the individual fluctuations of disease activity into account.^44^^,^^45^^,^^47^

In short, there is still a need of tests either broadly applicable to all patients, or applicable to complementary groups of patients covering the whole PSC continuum. The variations of scores and liver function tests in various subgroups of PSC patients is illustrated in Supplementary Figure S1. We compared the predictive performances of the MELD, revised MRS, Amsterdam–Oxford and PREsTO scores (Table 3). As expected, the c-statistics were similar and were above 0.73 both predicting transplant-free survival/hepatic decompensation at time of inclusion as well as by using repeated scores during the study period. Although these scores exhibit relatively good performances at the “cohort-scale”, individual prognostication remains more complex and factors such as the cholangiographic severity, fibrosis markers, co-morbidities, symptoms, and other complications are part of the clinical practice prognostication process and might thus gained to be included in future scores. Such datasets are available in the SUPRIM database and might be of interest for future developments.

**Table 3:** Predictive capacity of the model for end-stage liver disease (MELD) score, the revised Mayo risk score (MRS), the Amsterdam–Oxford score and the Primary Sclerosing Cholangitis Risk Estimate Tool (PREsTO) in patients with PSC, assessed at time of inclusion and with interpatient variation during follow-up, by cox proportional hazards regression and Harrell's C statistics.

	Hazard ratio (95% confidence interval)	Harrell's C
Model for end-stage liver disease (MELD) score at inclusion	1.08 (1.04–1.13)	0.66
MELD, yearly measurements during follow-up	1.24 (1.20–1.28)	0.80
Revised Mayo risk score (MRS) at inclusion	1.22 (1.10–1.35)	0.75
MRS, yearly measurements during follow-up	3.64 (2.83–4.68)	0.84
Amsterdam–Oxford score at inclusion	1.00 (1.00–1.00)	0.67
Amsterdam–Oxford score, yearly measurements during follow-up	4.37 (2.88–6.62)	0.84
Primary Sclerosing Cholangitis Risk Estimate Tool (PREsTO) at inclusion	1.02 (1.01–1.02)	0.72
PREsTO, yearly measurements during follow-up	1.05 (1.04–1.06)	0.79

Overall, the SUPRIM cohort captured the biological, clinical features and outcomes in PSC. It consequently offers an important resource for the development, validation or comparison or prognostic models.

## Discussion

Although the natural history of PSC remains largely unpredictable, disease progression at a cohort-scale and risk of LT, HB cancer or death is associated with age, UC, fibrosis stage and severity of bile duct changes.^14^^,^^49^^,^^52^ Relatively better long-term outcome has been reported in subgroups of patients such as small duct PSC and women.^12^ In this multicentre prospective cohort of unselected PSC patients, the clinical course was in general benign, regardless of underlying phenotype. PSC with concomitant IBD, represents one of the most well described phenotypes and approximately 70% of patients with PSC have a concomitant diagnosis of IBD, often preceding that of PSC.^14^^,^^53^^,^^54^ Poorer long-term outcome has been reported in patients with PSC-IBD, especially in patients with concomitant UC, suggesting that the progression of liver disease might be contingent on IBD subtype.^14^^,^^55^^,^^56^ In this study, the occurrence of IBD or its subtypes was not significantly associated with hepatic decompensation and/or severe complications of cholangitis, which might suggest that IBD phenotype is of marginal importance. Confirming previous studies, small duct PSC was associated with a more benign clinical course and seems more common when features of AIH is present.^14^^,^^57^ Elevated levels of IgG4, previously reported to be associated with worse outcome^18^ was found in a limited number of patients (1%), which is lower than previously reported.^58^ Unlike most autoimmune conditions PSC primarily affects men, however a recent report suggested a higher prevalence of PSC in women than has previously been described, undiagnosed due to a more quiescent clinical course.^12^^,^^59^ The prognostic scores (revised MRS, Amsterdam–Oxford; PresTo) performed over-all well in the SUPRIM cohort, assessed by c-statistics. As excepted, the predictive capacity was higher when repeated yearly as compared to calculated at time of inclusion. Importantly these scores reflect the cohort scale perspective and should therefore be used with caution on the patient scale level in clinical practise. This is of special importance since prognostication is one of the most common requested information from patients.^60^ As these results can certainly be improved, more work could be performed using datasets of the SUPRIM cohort, including AI based approaches, to establish new scores or validate others.

The SUPRIM cohort gathers both data and biological sample available today for research projects. It is a growing resource and some of the patients have reached 10 years follow-up. Several similar initiatives have been launched recently such as a Swiss cohort initiated in 2017 aiming to include 120 patients (Clinicaltrials.gov NCT03146936) and an Italian cohort initiated in 2022 with the aim to include 6000 patients with PSC (Clinicaltrials.gov NCT05618145) where biological samples are collected only at time of recruitment. The effort to collect longitudinal samples for a long period of time is huge. Not only the high cost is a challenge, but in our experience also the compliance for regular sampling, especially in non-advanced PSC patients reduce with time. Additional initiatives are ongoing with patients’ organisations (PSC Partners Seeking A Cure) and within the International PSC Study Group (https://www.ipscsg.org), as well as the Scandinavian PSC Biobank (Scand-PSC). Scand-PSC is a collaborative biobanking effort from Norway and Sweden, supported by Halloran Foundation, and can in Sweden serve as a continuum of the SUPRIM collection. In addition, we also perform collections of bile, peripheral cells and cytobrush in patients undergoing ERCP for clinical indications at our centre. Additional biological material (whole blood, serum, plasma, and faecal samples) is also collected from the SUPRIM cohort in the context of the PiSCATIN study, a phase III randomized double-blind controlled trial of 40 mg simvastatin versus placebo.^40^ This offers many opportunities of research projects including but not limited to the generation of new datasets with cutting edge technologies and targeted validation of promising discoveries to move towards implementation.^61^

Randomised control trials (RCT) in PSC have been historically difficult to conduct due to the rareness of the disease, its heterogeneity, its slow progression, and the lack of surrogate markers to evaluate drug efficacy. Drugs are classically tested in large groups of selected patients for a relatively long period of time often relying on observations of hard end points. As a result, few drugs have been tested as compared to the large portfolio potentially of interest. Although this approach might provide strong evidence, results are released at a relatively slow pace. Inclusion of more patients in shorter trials to unravel the potential of drug repurposing with the aim of implementing personalised therapies for PSC patients is warranted. As a prospective observational study of unselected PSC patients, the SUPRIM cohort provides both real world data (RWD) and real-world evidence (RWE). In this context, the SUPRIM cohort and available samples could be used to generate biological fingerprints hallmarks of natural history and progression of PSC phenotypes as well as a potential control arm for single arm trials. Such strategy has already been successfully used in the past and lead to drug approvals.^62^, ^63^, ^64^, ^65^, ^66^

Beside its relevance for clinical trials, the SUPRM cohort is a valuable tool for the development and validation of early diagnostic tests for HB cancer. Indeed, PSC is the highest known risk population for BTC, yet current strategies fail to detect these cancers early enough to improve the survival of patients.^41^ In this context, repeated sampling before the diagnosis of BTC might be of high relevance. Moreover, in addition of benefiting PSC patients, one might anticipate that such findings might also be useful for screening strategies of sporadic BTC in other populations.

We envision the SUPRIM cohort as an open-access collaborative resource to accelerate the generation of new knowledge and independent validations of promising ones. We therefore encourage people to contact us for project proposals.

## Contributors

Conceptualisation: AB, FR, EN, SK; data collection: AB, FR, EN, SK, EvS, AM; data curation: CV, MC; formal analysis MC, CV; funding acquisition: AB; Original draft: MC, CV; review and editing All authors.

## Data sharing statement

The authors confirm that data are available from the corresponding author (MC), upon reasonable request.

## Declaration of interests

The authors have no conflict of interest to disclose.

## Appendix A Supplementary data

Supplemenary Tables and Figures

Study protocol
