# Supplementary material for: The Swedish initiative for the study of Primary sclerosing cholangitis (SUPRIM)
Source: eClinicalMedicine. 2024 Mar 11;70:102526. doi: 10.1016/j.eclinm.2024.102526 (PMC10945116; doi:10.1016/j.eclinm.2024.102526)
Supplement: Supplemenary Tables and Figures [file mmc1.docx]

Supplementary material

Supplementary table 1. Participating centers and number of patients with PSC.

Supplementary figure 1a. Fluctuations of liver function tests and predictive scores in patients with and without LT.

Supplementary figure 1b. Fluctuations of liver function tests in patients with PSC with and without concomitant IBD.

Supplementary figure 1c. Fluctuations of liver function tests in patients with PSC with features of autoimmune hepatitis.

Supplementary figure 1d. Fluctuations of liver function tests in patients with small duct PSC.

Supplementary figure 1e. Fluctuations of predictive scores in patients with depending on duration of PSC (>10years), BMI (>30) and gender.

Supplementary table 1

Participating centres and number of patients with PSC per centres.

| **Participating hospitals** | | |
| --- | --- | --- |
|  | **PSC patients (512)** | |
| Danderyd Hospital | 11 | |
| Ersta Hospital | 10 | |
| Karolinska University Hospital | 205 | |
| Linköping University Hospital | 25 | |
| Sahlgrenska University Hospital | 93 | |
| Skåne University Hospital | 60 | |
| Stockholm South General Hospital | 11 | |
| Vrinnevi Hospital, Norrköping | 4 | |
| University Hospital of Umeå | 19 | |
| Uppsala University Hospital | 47 | |
| Örebro University Hospital | 27 |  |

Figure 1a. Fluctuations of liver function tests and predictive scores in patients with and without LT.

Figures 1b. Fluctuations of liver function tests in patients with PSC with and without concomittant IBD.

Figure 1c. Fluctuations of liver function tests in patients with PSC with features of autoimmune hepatitis.

Figures 1d. Fluctuations of liver function tests in patients with small duct PSC.

Figure 1e. Fluctuations of predictive scores in patients with depending on duration of PSC (>10years), BMI (>30) and gender.
