# Supplementary material for: The Swedish initiative for the study of Primary sclerosing cholangitis (SUPRIM)
Source: eClinicalMedicine. 2024 Mar 11;70:102526. doi: 10.1016/j.eclinm.2024.102526 (PMC10945116; doi:10.1016/j.eclinm.2024.102526)
Supplement: Study protocol [file mmc2.docx]

| **A Surveillance Study for Early Detection of Cancer in Primary Sclerosing Cholangitis**  ***2011-02-15*** | | |
| --- | --- | --- |
|  | Coordinating Investigator | Annika Bergquist, Gastrocentrum, Karolinska University Hospital |

Table of Contents

Summary 3

1 ABBREVIATIONS 4

2 AdministrativE information 5

3 BACKGROUND INFORMATION 5

Study Objectives 6

The objective of the current study is: 6

4 QUESTION 6

4.1 Primary question 6

5 Endpoints 6

6 Design 6

6.1 General design 6

6.2 Assessments and procedures 7

6.3 Flow chart 8

7 STUDY SUBJECTS 8

7.1 Inclusion criteria 8

7.2 Exclusion criteria 9

7.3 Criteria for withdrawal 9

8 StATISTICS AND DATA MANAGEMENT 9

8.1 Data management 9

9 EtHICS 9

9.1 The Swedish Ethical Review Authority 9

9.2 Ethical implementation of the study 9

9.3 Evaluation of risks and benefits 10

9.4 Study subjects’ information and informed consent 10

9.5 Case Report Forms 10

9.6 Archiving 10

10 PublicATION OF RESULTS 10

11 PROTOCOL AMENDMENTS 10

12 STAFF information 10

13 RefeRENCES 11

14 FlOWCHART 12

Summary

| **PROTOCOL TITLE AND OBJECTIVES** | |
| --- | --- |
| Protocol title: | A surveillance study for early detection of cancer in primary sclerosing cholangitis (PSC) |
| Study objectives: | To investigate whether surveillance with an annual magnetic resonance cholangiopancreatography imaging (MRI/MRCP) and modern endoscopic and radiological techniques can identify early cancer development in PSC and offer a cure for the cancer. |
|  |  |
|  |  |
| **STUDY SUBJECT POPULATION** |  |
| Study subjects: | Patients with PSC |
| Number: | 600 |
|  |  |
| **STUDY TIMELINE** |  |
| First patient included: | 30-05-2011 |
| Last patient included: | 30-04-2014 |
| Last patient completed: | 31-12-2019 |

ABBREVIATIONS

| **Abbreviation** | **Explanation** |
| --- | --- |
| CRF | Case Report Form |
| PSC | Primary Sclerosing Cholangitis |
| CC | Cholangiocarcinoma |
| CA19-9 | Carbohydrate Antigen 19-9 |
| CEA | Carcinoembryonic Antigen |
| FISH | Fluorescent in situ hybridization |
| IBD | Inflammatory Bowel Disease |
| PET | Positron Emission Tomography |
| FDG-PET | 2[^18^F] fluoro-2-deoxy-D-glucose Positron Emission Tomography |
| MRI | Magnetic Resonance Imaging |
| ERCP | Endoscopic Retrograde Cholangiopancreatography |
| MRCP | Magnetic Resonance Cholangiopancreatography |
| VAS | Visual analogue scale |

AdministrativE information

**Coordinating Investigator**

Karolinska University Hospital

Annika Bergquist

Gastrocentrum K63

141 86 Stockholm

[annika.bergquist@ki.se](mailto:annika.bergquist@ki.se)

**Other Responsible Investigators**

| Sangfelt Per,  Rorsman Fredrik | Department of Gastroenterology, Uppsala University Hospital, 751 85 Uppsala |
| --- | --- |
| Werner Mårten | Clinical Medicine, University Hospital of Umeå,  901 85 Umeå |
| Wallerstedt Sven | Clinical Medicine, Östra sjukhuset, 416 85 Gothenburg |
| Bergquist Annika,  Hultcrantz Rolf | Karolinska Huddinge and Solna, Gastrocentrum, K61, 141 86 Stockholm |
| Almer Sven  Lindgren Stefan | Experimental Medicine Clinic, 581 85 Linköping |
| Verbaan Hans,  Prytz Hanne | Gastroenterology and Internal Medicine, Skåne University Hospital, 205 02 Malmö |
| Nilsson Emma,  Friis-Liby Ingalill | Department of Gastroenterology, Skåne University Hospital,  221 85 Lund |
| Hanns Ulrich Marschall | Department of Gastroenterology, Sahlgrenska University Hospital 41345 Gothenburg |
| Nyhlin Nils | Department of Clinical Medicine, Örebro University Hospital,  701 85 Örebro |

BACKGROUND INFORMATION

Primary sclerosing cholangitis (PSC) is a chronic, cholestatic liver disease with an unknown aetiology and a prevalence of 8-14/100,000. The majority of cases are men and up to 80% have inflammatory bowel disease (IBD). With PSC the course varies. The disease can be asymptomatic but typical symptoms like jaundice with itching, cholangitis and tiredness can occur during the course of the disease and after 12-18 years, cirrhosis of the liver with liver failure has developed in half of the patients.

Patients with PSC have a high risk of developing cancer, both in the bile ducts and large intestine with concomitant IBD, but also in the pancreas. The risk of colorectal cancer is increased 10 times compared to the normal population and 5 times compared to patients with a healthy liver with extensive ulcerative colitis. The risk for colorectal cancer each year can be estimated to be approximately 2% (1). Due to the high cancer risk, annual colonoscopy checks in these patients are recommended and if dysplasia develops, patients are operated and a colectomy is performed. Cholangiocarcinoma (CC) is the most feared complication of PSC. The risk for CC has been estimated to be approximately 1.5% per year with a lifetime risk of up to 20% (2).

There is a lack of medical treatment that slows the course of the disease and reduces the risk of cancer and the only curative treatment if liver cirrhosis with complications has presented itself is a liver transplant. PSC is the most common single cause of liver transplantation in Scandinavia (3). After a liver transplant, the risk for colorectal cancer remains and there are a few individual reports that say it may even be increased (4). If CC has occurred, then often the patient cannot have a transplant due to a rapid recurrence and growth of cancer in the transplanted organ during ongoing immunosuppression. There are a few individual studies that suggest that transplantation early in the progress of CC, alone or in combination with external radiation, brachytherapy and cytostatics in accordance with an extremely strict protocol (so-called Mayo Protocol), can lead to a cure in some patients (5,6). Without treatment, survival after a CC diagnosis is 3-6 months.

Surveillance for CC with PSC (with analysis of CA 19-9 in combination with ultrasound, CT, MRI and/or ERC with brush cytology and FISH/DNA abberations) is controversial but in one study, has been reported to be able to diagnose CC early on and improve survival (7).

We have previously evaluated the diagnostic value of dynamic positron emission tomography (PET) with 2[^18^F]fluoro-2-deoxy-D-glucose (FDG) for CC in 24 PSC patients that were planned for liver transplantation (8). All patients had a preoperative transplantation analysis without a definite finding of malignancy and underwent a preoperative FDG-PET. PET diagnosis was compared with histopathology from explanted liver and FDG-PET had a positive predictive value with CC or CC in situ (= high grade dysplasia) at 75% and a negative predictive value of 95%.

Assuming the diagnostic sensitivity and specificity and the positive and negative predictive values of the previously reported investigation modalities for CC in an unselected material of PSC patients (9) it seems more cost-effective as an initial surveillance investigation to use MRI/MRC and S-CA 19-9. In case of suspected malignant findings, patients are investigated using ERC and/or PET-CT according to clinical practice. Modern investigations of bile ducts include cholangioscopy with the SpyGlass technique, which also enables biopsy from the bile ducts.

**Hypothesis**

Through annual surveillance with Magnetic Resonance Cholangiopancreatography (MRCP)/MRI + CA 19-9, we can identify early stage cholangiocarcinoma, thereby offering a potentially curative treatment.

Study Objectives

The objective of the current study is:

1. If surveillance with MRCP can identify early malignant changes in PSC.
2. Investigate the value of PET diagnostics in early malignant changes in bile ducts in patients with dominant strictures in PSC.
3. To evaluate yearly colonoscopic surveillance in PSC-IBD
4. To collect a prospective cohort with complete clinical data and biobanked samples for further research

QUESTION

Primary question

Using annual MRCP investigations and modern endoscopic and radiological techniques to identify early cancer development with PSC, is it possible to offer a cure against cancer? Can PET diagnostics of malignant changes in bile ducts in patients with dominant strictures in PSC contribute to diagnostic accuracy?

Endpoints

## Primary endpoints: Hepatobiliary cancer (CC, hepatocellular carcinoma, gall bladder carcinoma)

**Secondary endpoints:** Liver transplantation, death, colorectal cancer

Design

General design

Patients are followed annually by using clinical characteristics, symptom scores, other treatments, liver tests including CA 19-9, MRI, MRCP and coloscopy with biopsies. Patients are included either in connection with a colonoscopy or an MRI/MRCP (<2 months old). It is important that the surveillance investigations (coloscopy and MRI/MRCP) are performed regularly with a 1-year interval (± 2 months) but it is NOT important that a colonoscopy and MRI/MRCP are synchronised with each other. Upon inclusion, the date is indicated for the MRCP and colonoscopy, respectively and investigations are repeated annually according to these dates. MRCP surveillance is only performed in patients that have not undergone a liver transplant. Colonoscopy surveillance is only performed in patients with IBD. In the event a dominant stricture is identified, an ERCP is carried out using brush cytology and if this shows dysplasia, a PET-CT is performed if there is availability at the hospital and in line with local practice. Patients are recruited at gastroenterology clinics throughout Sweden and a total of 600 patients will be included. Follow-up and interventions (except for an annual MRI/MRCP), does not normally differ from the regular clinical follow-ups that are carried out at the clinic. Patients that are included in the study are recruited via their physician and study follow-up can be taken care of without interrupting the patient-doctor continuity.

Assessments and procedures

**Inclusion**

- Oral and written patient information
- Informed consent is obtained
- Verification of inclusion and exclusion criteria
- Details of age, gender and medical history is registered
- Clinical information about PSC and IBD is registered, including information about the most recent MRI/MRCP/colonoscopy
- VAS-scale (0-10) for estimation of itching
- Health examination
- Registration of ongoing illnesses/diseases
- Registration of concomitant medications
- Blood tests: Bilirubin, ASAT, ALAT, GT, ALP, Albumin, PK, INR, Creatinine, Na, K, CEA, CA19-9, Hb, TPK, LPK, amylase
- Serum – whole blood and plasma samples to be frozen for future analysis (for example, for tumour markers and bile acids)
- Autoantibodies (ANA, AMA, SMA, LKM1) Total IgG and IgA, IgG4, HBsAg, Anti-HCV

**Annual follow-up for five years** (12 months, 24 months, 36 months, 48 months, 60 months).

- Ongoing illnesses
- Concomitant medications
- Registration of the onset of symptoms, activity of IBD and interventions
- Results from the MRI/MRCP performed since the previous visit (copy attached to CRF) (± 2 months) (this also applies to small duct PSC)
- Results from any ERCP (ERCP is carried out only in the event of a follow-up visit, if necessary, i.e., if there are suspicious changes on the MRCP that need to be mapped out further with an ERCP)
- Results from a colonoscopy (± 2 months)
- Blood tests: Bilirubin, ASAT, ALAT, GT, ALP, Albumin, PK, INR, Creatinine, Na, K, CEA, CA19-9, Hb, TPK, LPK, amylase
- Details about cancer development
- Serum and plasma samples to be frozen for future analysis (for example, for tumour markers and bile acids)

In patients who undergo MRI/MRCP during the course of the study due to a clinical indication, an annual follow-up check is carried without MRCP, which is calculated after the most recent investigation.

**ERCP**

In the event of an indication of ERCP, a brush sample is always taken. Ideally at the same time as a similar test method to the brush cytology, see sampling instructions “Brush cytology with ERCP”

**Blood tests/Bio banking**

All blood tests are taken according to the protocol (see the flow chart) and are analysed at a local clinical chemistry laboratory and entered in the CRF. Extra research samples for future analyses are marked and handled according to special instructions “Labelling of samples” and “Manual for handling samples”. The samples are stored at each respective centre in a -70°C freezer and transported annually to the coordinating investigator together with a code key.

**MRCP**

Carried out at each respective centre, ideally according to a special protocol with Primovist.

**Follow-up** (7 and 10 years after inclusion, respectively)

- Details about the cancer development, colectomy, liver transplant and death will be collected

Flow chart

The management of patients concerning liver malignancy in the study is shown in the flowchart below. Also see the Appendix with flowchart detailing the test sampling and investigations.

Flow chart

Monitoring with MRI/MRCP annually

No tumour suspected stricture, new investigation in 1 year

Tumour suspect stricture with wall thickening and/or contrast upload in the wall

Unchanged

Progress

PET-CT (centre with availability)

Insufficient material didn’t come up

ERCP with brush cytology ± dilation/stent + FISH

Tumour mass

Benign

FISH positive/Malignant/Dysplasia/DNA aneuploidy

New MRCP 3-6 months

Spyglass with biopsy

Radiological staging of tumour including PET

Plan for therapy (resection, LTX, Chemo)

STUDY SUBJECTS

Inclusion criteria

- All patients with PSC and without IBD can be included in the study
- Men and women over 18 years of age
- Expected survival of more than 1 year
- Has previously undergone coloscopy and MRCP or ERCP
- Has given written consent to participate
- Transplanted people that have the colon remaining may be included in the study if other criteria are met but, in these cases, only colonoscopy surveillance is carried out. Colectomised patients may be included but then only with MRCP surveillance.

Exclusion criteria

- Patients on the waiting list for transplantation
- Recently transplanted patients with a follow-up period of less than 1 year
- Patients that are both transplanted and colectomised
- Secondary sclerosing cholangitis

NOTE! Patients with small duct PSC and overlap with autoimmune hepatitis may be included in the study if other criteria are met.

Criteria for withdrawal

- Patients have the right to withdraw their participation in the study at any time.

For patients that withdraw from the study prior to the end point, information about the reasons for withdrawal will be collected if the patient wants to respond to this.

After the end of the study, patients continue their treatment according to normal clinical routines.

StATISTICS AND DATA MANAGEMENT

Data management

Results from analyses and investigations are registered on specially prepared forms, CRF’s, where the patient is only identified with a code number. Data is registered, saved and later analysed in a database that the sponsor is responsible for. Code lists are stored at each respective investigator but the code key is transferred to the biobank at Karolinska University Hospital for centralising the information. All presentations of the data are carried out de-identified.

EtHICS

The Swedish Ethical Review Authority

The coordinating investigator is responsible for approval from the Swedish Ethical Review Authority. Approved by the regional Ethics Committee in Stockholm (Dnr 2011/824-31/2).

Ethical implementation of the study

The study will be carried out according to the study protocol, Good Clinical Practice (GCP), regulatory requirements and the Declaration of Helsinki.

Evaluation of risks and benefits

Patients with PSC have a high risk of developing cancer, both in the bile ducts and large intestine with concomitant IBD, but also in the pancreas. This occurs most commonly between 30-50 years of age as most people affected by PSC are young. The risk of colorectal cancer is increased by 10 times and in the liver 160 times, compared to the normal population (2). Due to the high risk of cancer, an annual colonoscopy check is recommended in these patients and if dysplasia develops, then patients are operated with a colectomy. Liver tumours are often detected late and there is often no treatment. The mean survival rate in the event of a liver cancer diagnosis is less than 6 months. Early diagnostics allow for several treatment possibilities that can lead to a cure or an extended life. The risks/discomfort that exist through study participation are minor. The potential benefit of the study results is deemed to far exceed the disadvantages associated with the study.

Study subjects’ information and informed consent

The investigator is responsible for providing oral and written information and receiving and signing consent forms with the patient. The originals must remain at the clinic and a copy is provided to the patient.

Case Report Forms

Data that is collected in the study will be registered in the Case Report Form (CRF), where the patient is only identified with a code number. The code key is stored with the investigator and is collected by the coordinating investigator throughout the entire study.

Archiving

The study documentation must be archived for a minimum of 10 years after the study report has been submitted to the Swedish Medical Products Agency. The document must be archived in a legible condition for any potential future audits or inspections.

PublicATION OF RESULTS

The plan is to publish results from the study in peer-reviewed scientific journals.

PROTOCOL AMENDMENTS

Any significant amendments to the protocol that are not purely administrative require approval from the Swedish Ethical Review Authority before they may be implemented.

STAFF information

The principal investigator at each respective study centre is responsible for ensuring that everyone working on the study is well informed and can perform the relevant elements of the study.

RefeRENCES

1. Broome U, Bergquist A. Primary sclerosing cholangitis, inflammatory bowel disease and colon cancer. Semin Liver Dis. 2006 Feb;26(1):31-41. Review

2. A Bergquist, A Ekbom, R Olsson, D Kornfelt, L Lööf, Å Danielsson, R Hultcrantz, S Lindgren, H Prytz, H Sandberg-Gertzén, S Almer, U Broomé. Hepatic and extrahepatic malignancies in primary sclerosing cholangitis. Journal of Hepatology 2002; 36:321-327.

3. Brandsaeter B, Isoniemi H, Broomé U, Olausson M, Bäckman L, Hansen B, Schrumpf E, Oksanen A, Ericzon BG, Höckerstedt K, Mäkisalo H, Kirkegaard P, Friman S, Bjøro K. Liver transplantation for primary sclerosing cholangitis; predictors and consequences of hepatobiliary malignancy. J Hepatol. 2004 May;40(5):815-22.

4. Dvorchik I, Subotin M, Demetris AJ. et al. Effect of liver transplantation on inflammatory bowel disease in patients with primary sclerosing cholangitis. Hepatology 2002; 35: 380-384

5. Boberg KM, Jebsen P, Clausen OP, Foss A, Aabakken L, Schrumpf E. Diagnostic benefit of biliary brush cytology in cholangiocarcinoma in primary sclerosing cholangitis. J Hepatol. 2006 Oct;45(4):568-74. Epub 2006 Jun 21

6. Rosen CB, Heimbach JK, Gores GJ. Liver transplantation for cholangiocarcinoma. Transpl Int. 2010 Jul;23(7):692-7.

7. Charatcharoenwitthaya P, Enders FB, Halling KC, Lindor KD. Utility of serum tumor markers, imaging, and biliary cytology for detecting cholangiocarcinoma in primary sclerosing cholangitis. Hepatology 2008; 48: 1106-1117).

8. Prytz H, Keiding S, Björnsson E, Broomé U, Almer S, Castedal M, Munk OL; Swedish Internal Medicine Liver Club. Dynamic FDG-PET is useful for detection of cholangiocarcinoma in patients with PSC listed for liver transplantation. Hepatology. 2006 Dec;44(6):1572-80.

9. Charatcharoenwitthaya P, Enders FB, Halling KC, Lindor KD. Utility of serum tumor markers, imaging, and biliary cytology for detecting cholangiocarcinoma in primary sclerosing cholangitis. Hepatology. 2008 Oct;48(4):1106-17.

FlOWCHART

|  | **Inclusion** | **12 months** | **24 months** | **36 months** | **48 months** | **60 months** (or at the end of the study if endpoint is reached) |
| --- | --- | --- | --- | --- | --- | --- |
| Informed consent | X |  |  |  |  |  |
| Inclusion/exclusion criteria | X |  |  |  |  |  |
| Randomisation | X |  |  |  |  |  |
| Liver function tests, CA 19-9, CEA | X | X | X | X | X | X |
| IgG4  Autoantibodies  IgG, IgA  HBsAg, Anti-HCV | X |  |  |  |  |  |
| Colonoscopy | X | X | X | X | X | X |
| MRI/MRCP | X | X | X | X | X | X |
| List of medications | X | X | X | X | X | X |
| Clinical information according to CRF | X | X | X | X | X | X |
| Serum and plasma for freezing | X | X | X | X | X | X |
| Whole blood for freezing | X |  |  |  |  |  |
